# A mutant screening method by critical annealing temperature-PCR for site-directed mutagenesis

**DOI:** 10.1186/1472-6750-13-21

**Published:** 2013-03-11

**Authors:** Ying Liu, Ting Wu, Jian Song, Xuelian Chen, Yu Zhang, Yu Wan

**Affiliations:** 1 Department of Physiology, School of Basic Medical Sciences, Wuhan University, Hubei, 430071, People’s Republic of China; 2 Center for Medical Research, Wuhan University, Hubei, 430071, People’s Republic of China; 3 Department of Anatomical Sciences, School of Basic Medical Sciences, Wuhan University, Hubei, 430071, People’s Republic of China; 4 Department of Gastroenterology, Zhongnan Hospital, Wuhan University, Hubei, 430071, People’s Republic of China

**Keywords:** Mutant screening, *Dpn* I digestion, Critical annealing temperature-PCR, Site-directed mutagenesis

## Abstract

**Background:**

Distinguishing desired mutants from parental templates and undesired mutants is a problem not well solved in Quikchange™ mutagenesis. Although *Dpn* I digestion can eliminate methylated parental (WT) DNA, the efficiency is not satisfying due to the existence of hemi-methylated DNA in the PCR products, which is resistant to *Dpn* I. The present study designed a novel critical annealing temperature (*T*_c_)-PCR to replace *Dpn* I digestion for more perfect mutant distinguishing, in which part-overlapping primers containing mutation(s) were used to reduce initial concentration of template DNA in mutagenic PCR. A *T*_c_-PCR with the same mutagenic primers was performed without *Dpn* I digestion. The *T*_c_ for each pair of the primers was identified by gradient PCR. The relationship between PCR-identified *T*_c_ and *T*_m_ of the primers was analyzed and modeled with correlation and regression.

**Results:**

Gradient PCR identified a *T*_c_ for each of 14 tested mutagenic primers, which could discriminate mismatched parental molecules and undesired mutants from desired mutants. The PCR-identified *T*_c_ was correlated to the primer’s *T*_m_ (r = 0.804, P<0.0001). Thus, in practical applications, the *T*_c_ can be easily calculated with a regression equation, *T*_c_ = 48.81 + 0.253*T_m_.

**Conclusions:**

The new protocol introduced a novel *T*_c_-PCR method for mutant screening which can more efficiently and accurately select against parental molecules and undesired mutations in mutagenic sequence segments.

## Background

PCR-based one-step site-directed mutagenesis (SDM) is an efficient and rapid method to generate gene mutation(s) for study of protein structure-function relationship, identification of gene expression and modification of vector [1-3]. Since established by Agilent Technologies according to Papworth *et al*. [4] and Nelson *et al*. [5], the QuikChange™ Site-Directed Mutagenesis System has been modified by a number of authors to make its procedure more simple and operable [6-11]. The principle of this technique is taking the double-stranded DNA of a target gene as the template and using a pair of overlapping oligonucleotide primers containing desired mutation to produce mutants by PCR. There are two concerns about this technology. Firstly, the number of cycles during PCR should be reduced to prevent undesired mutations; the resultant lower product yield can be offset by increasing the starting template concentration. Secondly, the parental (WT) DNA existing in final PCR products must be removed. For this, a mutant selection by digestion of *Dpn* I endonuclease is usually performed after PCR. However, although *Dpn* I (target sequence 5-GA^m^TC) can quickly digest fully methylated DNA (parental strands from bacterial strains), its reaction with hemi-methylated DNA (parental strand combined with PCR-generated strand) is 60-fold slower. Thus the final PCR mixture inevitably contains small amount of parental molecules. In addition, *Dpn* I digestion cannot select against undesired mutations. Here, the present study designed a novel critical annealing temperature (*T*_c_)-PCR method for more perfect mutant distinguishing, which can be used without *Dpn* I digestion. The *T*_c_ is derived from a regression equation, which can select against parental molecules up to a rate of 100% and undesired mutations located in mutagenic sequence segments.

## Methods

### Plasmid template

The plasmids used as templates for PCR were constructed using pET20b (+) (69739-3; Novagen, Germany) and pcDNA3.1(+) (V790-20, Invitrogen). The cDNA of different genes with different length, including human growth hormone (hGH) without signal peptide (NM_000515.3; 573 bp), hGH 2 variant isoform 3 precursor (hGH2V3, NM_022558.3; 738 bp), hGH receptor (hGHR; NM_000163.4, 1920 bp), porcine GH binding protein (pGHBP, NM_214254.2, 570 bp) or hGH binding protein (hGHBP, NM_000163.4, 714 bp), was inserted into either pET20b (+) between *Nde* I and *Hind* III, or pcDNA3.1 (+) between *Pst* I and *Eco* RI. The plasmids were propagated in *E. coli* DH5alpha cells (Agilent Technologies, CA) and isolated using Qiagen miniprep kits (Qiagen, Germany).

### Primers and site-directed mutagenesis

The primers for mutagenesis by PCR were designed basically according to the manufacturer (QuikChange™ Mutagenesis kit; Agilent Technologies, CA) but a modification was made according to Braman, *et al.*[4] and Liu, *et al.*[6] to reduce initial concentration of template DNA. Briefly, each pair of primers contained a non-overlapping sequence at the 3^′^-terminus and a primer-primer complementary (overlapping) sequence at the 5^′^-terminus. The non-overlapping sequence was significantly larger than the overlapping to make the melting temperature of the former higher than that of the latter. The designed primers listed in Table 1 were used for mutagenesis of the target residues D112G, D112K, I4V, L45D, E56D, R64D, R64M, N109Y and D116F in hGH molecule. All mutation sites were located in the complementary region, 3-7 bases away from the 5^′^-terminus. Thus, both forward and reverse primers shared the mutagenic region, but held a 9-17 base-long overlapping sequence. The total length of the primers varied from 21 to 38nt. The melting temperature (*T*_m_) was calculated using the formula provided by the manufacturer Agilent Technologies: *T*_m_ *= 81.5+ 0.41(%GC)-675/N - % mismatch.* Here, *N* is the primer length in bases. All the primers were synthesized by Generay Biotechnology (Shanghai, China). Mutagenic reaction was performed in 50 ul of PCR mix containing 2 ng of pET20b (+)-hGH (WT) as template, 200 nM primer and 2.5 U Pfu DNA polymerase (Fermentas, Canada). The PCR temperature profile was: an initial denaturation at 94°C for 3 min, followed by 30 cycles with each at 94°C for 30 sec, 55°C for 30 sec and 72°C for 0.5 kb/min, and a final extension at 72°C for 5 min. The PCR products of SDM were transformed into *E. coli* DH5alpha competent cells.

**Table 1:** The primers for site-directed mutagenesis

**Primer**	**Sequence**	**Length**	**Mismatches**	***T***_**m**_
**D112G(F)**	CTATGgCCTCCTAAAGGACCTAGAGGAAG	29	1	76.0
**D112G(R)**	GGAGGcCATAGACGTTGCTGTCAGAG	26	1	75.3
**D112K(F)**	CTATaAaCTCCTAAAGGACCTAGAGGAAG	29	2	68.3
**D112K(R)**	GGAGtTtATAGACGTTGCTGTCAGAG	26	2	66.8
**I4V(F)**	CAACCgTTCCCTTATCCAGGCTTTTTG	27	1	72.5
**I4V(R)**	GAAcGGTTGGGAACATATGTATATCTCCTTCT	32	1	73.9
**L45D(F)**	CATTCgatCAGAACCCCCAGACCTCCCTCTGTTTCT	36	3	76.1
**L45D(R)**	GGTTCTGatcGAATGAATACTTCTGTTCCTTTGGGAT	37	3	71.8
**E56D(F)**	TCAGAcTCTATTCCGACACCCTCCAAC	27	1	74.1
**E56D(R)**	GAATAGAgTCTGAGAAACAGAGGGAGGTCTG	31	1	76.3
**R64D(F)**	CTCCAACgacGAGGAAACACAACAG	25	3	63.8
**R64D(R)**	CCTCgtcGTTGGAGGGTGTCGGAATAG	27	3	69.7
**N109Y(F)**	CAGCtAtGTCTATGACCTCCTAAAGGACCTAGAGGAAG	38	2	77.9
**N109Y(R)**	GACaTaGCTGTCAGAGGCGCCGTACACCAG	30	2	76.9
**D116F(F)**	AAAGttCCTAGAGGAAGGCATCCAAACGCTGAT	33	2	73.6
**D116F(R)**	CTCTAGGaaCTTTAGGAGGTCATAGACGTTGCTGTCAG	38	2	77.9
**R64M(F)**	TCCAACAtGGAGGAAACACAACAG	24	1	68.0
**R64M(R)**	TCCaTGTTGGAGGGTGTCGGAATAG	25	1	71.8
**hGH2V3-P233S(F)**	TGGtCgTGGAAGgTGCTACTCCAGTGCCCACCAGCC	36	3	80.6
**hGH2V3-P233S(R)**	GAGTAGCAcCTTCCAcGaCCAGGAGAGGCACTGGG	35	3	79.4
**hGHBP-S237C(F)**	GATGtGCTAAGAATTCGAGCTCCGTCGACAA	31	1	76.3
**hGHBP-S237C(R)**	TTAGCaCATCTGAGGAAGTGTTACATAGAGCACC	34	1	76.8
**pGHBP-S237C(F)**	GATGtGCTAAGAATTCGAGCTCCGTCGACAA	31	1	76.3
**pGHBP-S237C(R)**	TTAGCaCATCTGAGGAAGTGTTACATAGAGCACC	34	1	76.8
**hGHR1-255R235C(F)**	AACAAtgcAACTCTGGAAATTATGGC	26	3	59.8
**hGHR1-255R235C(R)**	TTgcaTTGTTTGGATCTCACA	21	3	58.7
**hGHR1-638R235C(F)**	AACAAtgcAACTCTGGAAATTATGGC	26	3	59.8
**hGHR1-638R235C(R)**	TTgcaTTGTTTGGATCTCACA	21	3	58.7

*T*_m_ is the melting temperature of mutagenic oligonucleotide primers annealing with the WT template to 50%.

For all primers, the mutagenesis oligonucleotides are denoted in lowercase.

### Mutant screening by gradient PCR

For a more efficient mutant screening, a *T*_c_-PCR with the SDM-generated mutants as templates and the same mutagenic primer pairs was designed to replace the traditional *Dpn* I digestion. The *T*_c_, which can select against mismatched parental and undesired mutant templates from completely matched desired mutants was identified by a set of gradient-PCR with the generated clones from SDM and the same primer pairs listed in Table 1. Five clones were randomly collected from the transformants of each mutant and underwent PCR with the WT template as criteria. Six different annealing temperatures were designed according to the *T*_m_ of the primers bound to the WT template. Among these 6 tested temperatures, the annealing temperature at which there was still substantial amplification of the mutant template but no detectable products of the WT template was identified as the *T*_c_. The PCR was performed in a final volume of 20 μl PCR mix containing 20 ng template, 200 nM of each primer, 2 μl 10×reaction buffer (37.5 mM Tris-HCl, 10 mM (NH_4_)_2_SO_4_, 2 mM Mg^2+^, 0.01% Tween20) and 2U Taq DNA polymerase. The temperature profile was: initial denaturation at 94°C for 3 min followed by 35 cycles with each at 94°C for 30 sec, annealing at the desired temperature for 30 sec, extension for 2 kb/min and a final extension at 72°C for 5 min. The PCR products were then electrophoresed in 0.8% agarose gel to check their abundance. The clones that maintained detectable PCR products up to *T*_c_ or over were assumed as desired mutants and those that lost detectable products, the parental molecules. The results were then confirmed by DNA sequencing. Data of sequences were analyzed by the software Genetool lite version 1.0.

To evaluate the accuracy of the gradient-PCR identified *T*_c_ in distinguishing WT from mutant, a series of *T*_c_-PCR screening were performed with different target genes(hGH-D112K, hGH-D112G, hGH2V3-P233S, hGHR-R255C, hGHBP-S237C and pGHBP-S237C) after SDM, and the results were confirmed by sequence analysis.

### Colony gradient PCR

Colony PCR method is more widely used than plasmid PCR method in confirming the gene insertion into a vector. To test the feasibility of the *T*_*c*_-PCR used as a colony PCR, a portion of bacterial colonies containing the desired plasmid were picked using a sterile toothpick. The tip of the toothpick was placed in 180 ul of the PCR mixture and gently shaken. The PCR mix was then aliquoted into 25 ul aliquots. PCR was carried out using the gradient PCR protocol described above except that the initial denaturation time increased to 7 minutes.

### Data analysis

Data were statistically analyzed with SPSS version 18.0 (SPSS Inc., IL). A correlation analysis was performed to assess whether and how strongly the gradient PCR-identified *T*_c_ is related to *T*_m_ and other features of the primers (including the length, mismatch, %GC and the number of bases from 3^′^- or 5^′^-terminus to the mutation site). A stepwise linear regression was used to model the relationship between *T*_c_ and *T*_m_.

## Results

### Critical annealing temperature identified by gradient-PCR

**Figure 1:**
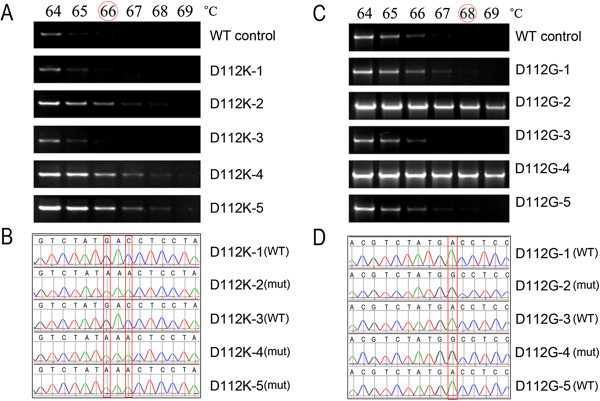
**Gradient PCR screening and sequence analysis of the transformants generated by SDM without *Dpn *I digestion. A**. Gradient PCR screening of multiple-site-mutant D112K. Five transformants were examined with the WT template as criteria under a set of gradient annealing temperatures from 64°C to 69°C. The identified *T*_c_ was 66°C at which the WT template lost detectable products. **B**. Sequence analysis of the same transformants, which shows that three transformants (D112K-2, 4 and 5) still detectable at annealing temperatures over *T*_c_ were desired mutants. **C**. Gradient PCR screening of single-site-mutant D112G. The identified *T*_c_ was 68°C. **D**. Sequence analysis of the same transformants showing that two transformants (D112G-2 and 4) still detectable at annealing temperatures over *T*_c_ were desired mutants.

Figure 1A showed the results of mutant screening by gradient PCR using D112K forward and reverse primers which contained two designed mutations. At the gradient annealing temperatures from 64 ~ 69°C, the WT template, as well as two clones (D112K-1 and D112K-3) out of five randomly collected from the transformants without *Dpn* I digestion, showed detectable products only at annealing temperatures of 64 and 65°C, whereas the other three clones (D112K-2, D112K-4 and D112K-5) maintained detectable up to 67°C or higher. Thus the *T*_c_ of D112K was identified as 66°C. Sequence analysis (Figure 1B) demonstrated that the two clones, D112K-1 and D112K-3, which lost detectable products at *T*_c_ (66°C) were WT molecules and the other three were the desired mutants (GAC was changed into AAA).

Figure 1C showed the gradient PCR screening for a single-site-mutant, D112G. The WT template and three clones (D112G-1, D112G-3 and D112G-5) lost detectable products at 68°C, whereas the other two (D112G-2 and 4) maintained detectable over 68°C; this annealing temperature was therefore identified as the *T*_c_ of D112G. Similarly, DNA sequence analysis demonstrated that the three clones becoming undetectable at the *T*_c_ were WT molecules and the other two (D112G-2 and 4) still detectable at or over *T*_c_ were desired mutants.

The gradient-PCR identified *T*_c_ for all tested primers were listed in Table 2.

**Table 2:** **Features of the primers and their gradient-PCR identified-*T***_**c**_

**Primer**	**Length**	**Mismatches**	**%GC**	Nt3*	**Nt5**^**Δ**^	***T***_**m**_	***T***_***c***_
**D112G(F)**	29	1	51.7	23	5	76.0	68
**D112G(R)**	26	1	57.7	20	5	75.3	68
**D112K(F)**	29	2	41.4	22	4	68.3	66
**D112K(R)**	26	2	46.2	19	4	66.8	66
**I4V(F)**	27	1	48.1	21	5	72.5	67
**I4V(R)**	32	1	40.6	28	3	73.9	67
**L45D(F)**	36	3	52.8	28	5	76.1	67
**L45D(R)**	37	3	40.5	27	7	71.8	67
**E56D(F)**	27	1	51.9	21	5	74.1	67
**E56D(R)**	31	1	48.4	23	7	76.3	67
**R64D(F)**	25	3	52.0	15	7	63.8	65
**R64D(R)**	27	3	59.3	20	4	69.7	65
**N109Y(F)**	38	2	47.4	31	4	77.9	69
**N109Y(R)**	30	2	60.0	24	3	76.9	69
**D116F(F)**	33	2	45.5	27	4	73.6	69
**D116F(R)**	38	2	52.0	29	7	77.9	69
**R64M(F)**	24	1	47.4	16	7	68.0	67
**R64M(R)**	25	1	40.5	21	3	71.8	67
**hGH2V3-P233S(F)**	36	3	63.9	19	3	80.6	70
**hGH2V3-P233S(R)**	35	3	62.9	17	4	79.4	70
**hGHBP-S237C(F)**	31	1	48.4	26	4	76.3	69
**hGHBP-S237C(R)**	34	1	44.1	28	5	76.8	69
**pGHBP-S237C(F)**	31	1	48.4	26	4	76.3	69
**pGHBP-S237C(R)**	34	1	44.1	28	5	76.8	69
**hGHR1-255R235C(F)**	26	3	38.5	18	5	59.8	60
**hGHR1-255R235C(R)**	21	3	38.1	16	2	58.7	60
**hGHR1-255R235C(F)**	26	3	38.5	18	5	59.8	60
**hGHR1-255R235C(R)**	21	3	38.1	16	2	58.7	60

* The number of bases from 3^′^- terminus to the mutation site.

Δ The number of bases from 5^′^-terminus to the mutation site.

### Mutant selection against WT and undesired mutants by critical annealing temperature PCR

**Figure 2:**
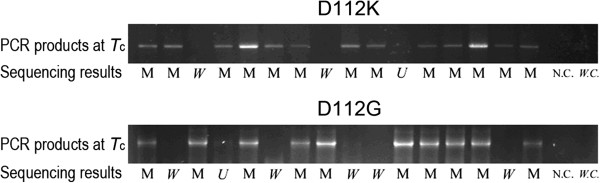
***T***_**c**_**-PCR screening and sequence analysis on 16 clones of D112K and D112G after SDM.** The *T*_c_ is 66°C for D112K and 68°C D112G. The sequencing results are represented by M (desired mutant), W (wild type) and U (undesired mutant), respectively. N.C and W.C represent negative and WT controls, respectively.

 Figure 2 showed the results of PCR screening with the gradient-PCR-identified *T*_c_ as annealing temperature and sequence analysis on 16 transformants of D112K and D112G after SDM without *Dpn* I digestion. At annealing temperature of *T*_c_ (66°C for D112K and 68°C for D112G), the clones that showed substantial PCR products were all desired mutants as demonstrated by DNA sequencing; whereas those that lost detectable products were either WT or undesired mutants that had “mistake” located in mutagenic sequence segments.

*T*_c_-PCR screening and sequence analysis on all tested clones of D112K and D112G were shown in Table 3.

**Table 3:** ***T***_**c**_**-PCR screening and sequence analysis on clones of D112K and D112G**

**Primers**	***T***_**c (°C)**_	**Tested clones**	***T***_**c**_**-PCR and sequence analysis**							
			***T***_**c**_**-PCR detect- able**	**Sequencing**			***T***_**c**_**-PCR undetect-able**	**Sequencing**		
				**Desired mut.**	**WT**	**Undesired mut.**		**Desired mut.**	**WT**	**Undesired mut.**
**D112K**	66	49	38	38	0	0	11	0	10	1
**D112G**	68	50	26	26	0	0	24	0	23	1

### Reliability of critical annealing temperature PCR at different initial concentrations of template

**Figure 3:**
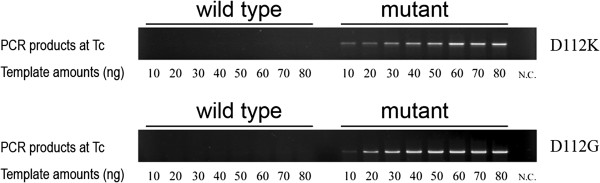
***T***_**c**_**-PCR with different amounts of hGH WT, and mutants D112K and D112G as templates.**

To test the reliability of *T*_c_-PCR at different initial concentrations of template, both WT and mutant plasmids (10-80 ng in 50 ul reaction volume) of D112K and D112G were used for PCR with *T*_c_ as annealing temperature. As shown in Figure 3, in the WT templates no detectable products could be observed at all tested concentrations, but in the mutant templates the PCR bands were detectable at 10 ng, and enhanced with concentrations from 10 to 50 or 60 ng.

### Relationship between gradient PCR-identified *T*_c_ and *T*_m_

The correlation analysis on relationship of gradient PCR-identified *T*_c_*to T*_m_ and other features of the primers (Table 2) showed that the PCR-identified *T*_c_ of 9 tested primer pairs of hGH mutants were correlated with their *T*_m_ (r = 0.804, P<0.0001). The relationship between *T*_c_ and *T*_m_ were statistically modeled with the stepwise linear regression. Taking *T*_m_ as the explanatory variable, the linear regression model for *T*_c_ and *T*_m_ was expressed as the following regression equation:

*T*_c_ = 48.81 + 0.253* T_m_*,* here, *T*_m_ is the melting temperature of mutagenic primer pairs with WT template.

**Figure 4:**
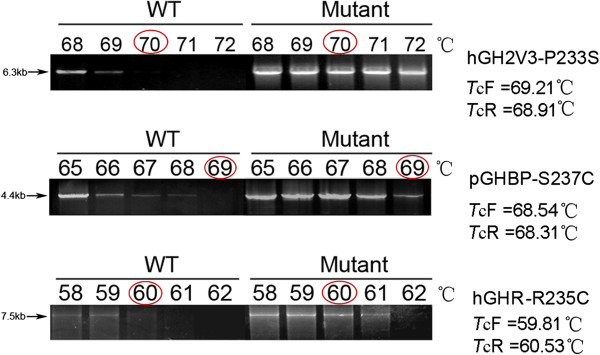
**Verification of regression equation-derived *T***_**c **_**by gradient PCR with different genes.** As showed in the figure, the *T*_c_ identified by gradient PCR (hGH2V3-P233S: 70°C, pGHBP-S237C: 69°C and hGHR-R235C: 60°C) were very close to those derived from the regression equation (hGH2V3-P233S: *T*_cF_ =69.21 and *T*_cR_ =68.91; pGHBP-S237C: *T*_cF_ = 68.54°C and *T*_cR_ = 68.31°C; and hGHR-R235C: *T*_cF_ = 59.81°C and *T*_cR_ = 60.53°C). Although hGHR-R235C was inserted into pcDNA3.1(+) (5428 bp) differing from pET20b(+) (3716 bp), the *T*_c_ from PCR was almost same as that from the equation predicted.

To test the repeatability of the regression equation-derived *T*_c_,a series of gradient PCR were run with different target genes (hGH-D112K, hGH-D112G, hGH2V3-P233S, hGHR-R255C, hGHBP-S237C and pGHBP-S237C). As a representative, hGH2V3-P233S contained 3 designed mutations. According to the regression equation above, the predicted *T*_c_ should be: *T*_cF_ (forward primer) = 69.21°C and *T*_cR_ (reverse primer) = 68.91°C. Thus, a set of annealing temperatures were selected from 68 to72°C. As shown in Figure 4 The WT template (pcDNA3.1- hGH2V3) lost detectable products at 70°C, whereas the mutant template (pcDNA3.1-GH2V3-P233S) maintained substantial products at 70°C. The PCR-identified *T*_c_**(**70°C**)** was very close to the equation predicted (*T*_cF_ = 69.21°C and *T*_cR_ = 68.91°C). Similarly,in the tests using other different genes with different length, the PCR-identified *T*_c_ were also very close to the equation predicted *T*_c_ (shown in Figure 4).

### Critical annealing temperature PCR screening with bacterial colonies as templates or with different sources of Taq DNA Polymerase

**Figure 5:**
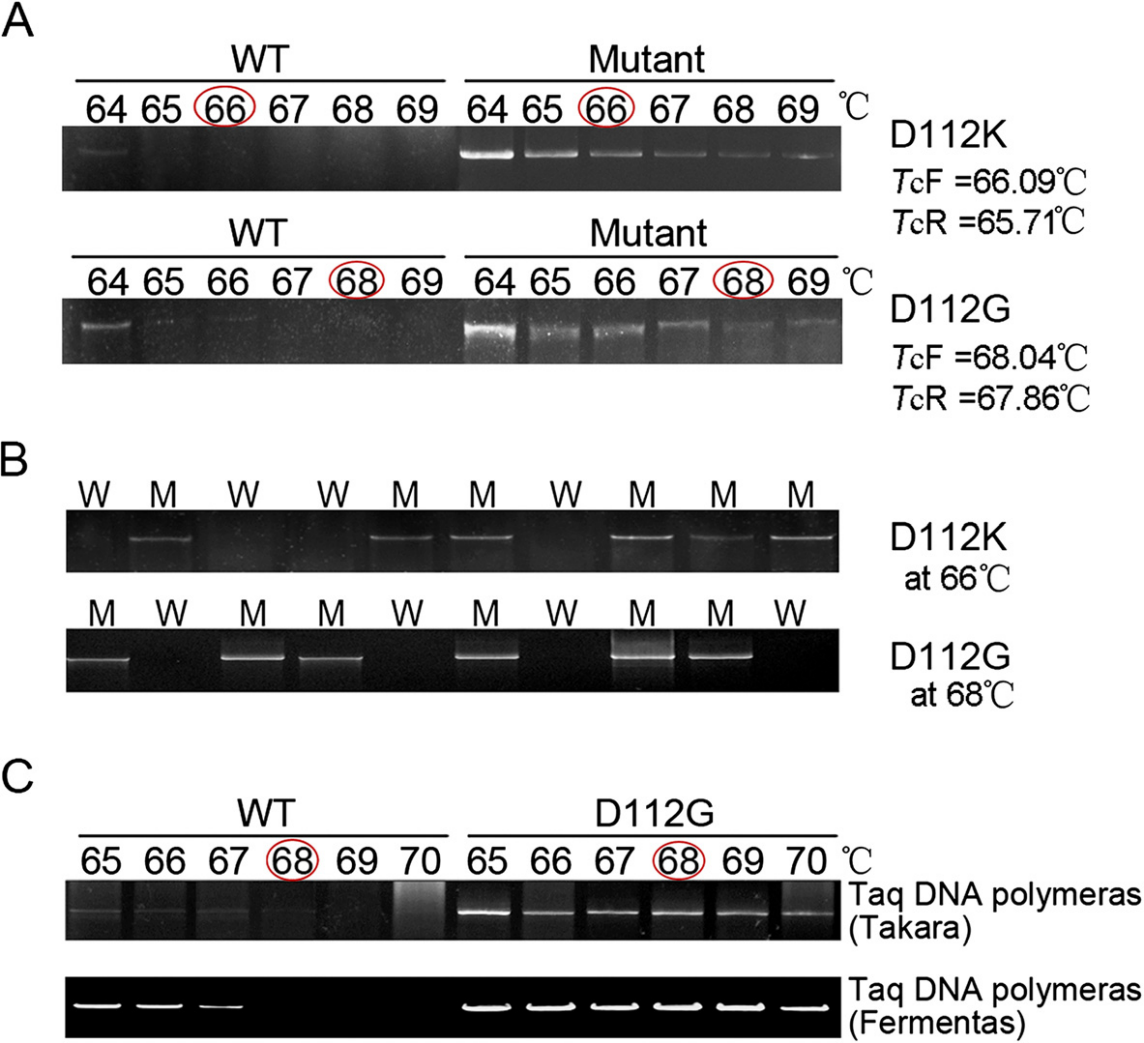
***T***_**c**_**-PCR screening with bacterial colonies as templates or with Taq DNA polymerase from different sources. A**. Colony gradient PCR performed at indicated annealing temperatures using bacterial colonies carrying hGH WT or hGH mutant (D112K or D112G) plasmid to detect their *T*_c_. **B**. Twenty colonies screened at *T*_c_ after SDM. The sequencing results are represented by M (desired mutant) and W (wild type), respectively. **C**. Gradient PCR at the indicated annealing temperatures with Taq DNA Polymerases from different companies, such as Fermentas and Takara.

When using the bacterial colonies containing the desired plasmid (PET20b(+)/hGH-D112K and -D112G) as templates, a *T*_*c*_ could be identified by a gradient PCR and the PCR-identified *T*_c_ (66°C and 68°C ) were also very close to the equation predicted (D112K: *T*_cF_ = 66.09°C and *T*_cR_ = 65.72°C; D112G; *T*_cF_ = 68.04°C and *T*_cR_ = 67.86°C) (Figure 5 and Table 2)*.* However, the present experiment suggests that the initial denaturation time should be increased to 7 min to enhance denaturation of the plasmids in *E. coli* DH5alpha cells.

 A similar result was also obtained when we repeated the study with the enzymes from different batches or different companies, such as recombinant Taq DNA Polymerase (Fermentas, Canada), Taq DNA Polymerase (Takara, Japan), and 2 × Taq master mix (Dsbio, Peking).

## Discussion

Distinguishing desired mutants from parental template and undesired mutations in SDM is still a problem not well solved. Although as a favorite method, digestion of *Dpn* I can eliminate fully methylated WT DNA (parental strands from bacterial strains), around 20-30% of hemi-methylated WT molecules (parental strand combined with PCR-generated strand) could not be removed [6,8] due to hemi-methylated DNA in the PCR products is more resistant to *Dpn* I [3,12]. In addition, *Dpn* I cannot select against undesired mutations.

Liu and Naismith [6] recently presented a strategy to minimize the parental molecules by enhancing the PCR efficiency. In their design scheme, each pair of primers contained non-complementary sequences at their 3^′^ terminus and primer-primer complementary sequences at the 5^′^ terminus. The mutation sites were placed in the complementary region. The non-overlapping sequences were longer than the complementary sequences so that they have a *T*_m_ higher than that of primer-primer complementary sequences. Thus the primer dimerization was eliminated and the new synthesized PCR products could be used as the templates in the subsequent PCR cycles. This design can significantly increase the PCR efficiency and requires less template DNA.

On the basis of Liu and Naismith, we designed a modified protocol in which the *Dpn* I digestion was replaced by a *T*_c_-PCR that could select against parental molecules and undesired mutations in mutagenic sequence segments at an accuracy of 100%. In this PCR the primers were the same used to introduce the mutations and the templates were SDM-generated clones. Thus theoretically the primers completely match the desired mutant and should have an annealing temperature to the mutant higher than to any other mismatched templates such as parental molecules and undesired mutants. Since DNA chemosynthesis is much more error-prone than DNA replication by polymerase [13-16], it is important to eliminate the undesired mutants generated by mistake primers. The key for this PCR screening is a critical annealing temperature (*T*_c_) that can discriminate mismatched templates from the matched desired mutant. Using a gradient PCR, we identified the *T*_c_ for each mutant template and demonstrated that it was correlated to the primer’s *T*_m_. Thus, in practical applications, the *T*_c_ can be easily calculated with a regression equation, *T*_c_ = 48.81 + 0.253* T_m_.

The reliability of the *T*_c_-PCR screening was confirmed by using different vectors harboring different genes (ranging from 573 bp to 1920 bp) and by employing Taq polymerases from different sources. Furthermore, the feasibility of the *T*_c_-PCR as a colony PCR was tested with bacterial colonies containing the desired plasmid. These suggest that the *T*_c_-PCR can have a wider usage.

## Conclusions

 In summary, the new protocol presented in the present study introduced a *T*_c_-PCR screening to replace the *Dpn* I digestion, which can more efficiently and accurately select against parental molecules and undesired mutants which have a “mistake” in mutagenic sequence segments.

## Abbreviations

WT: Wild type; SDM: Site-directed mutagenesis system; Tc-PCR: Critical annealing temperature-PCR.

## Competing interests

The authors have declared that no competing interests exist.

## Authors’ contributions

Y L designed the study, carried out all experiments, acquired and analyzed the data, drafted and revised the manuscript; T W analyzed the data and performed the statistical analysis; X C and Y Z carried out the site-directed mutagenesis and gradient PCR; J S participated in the design of the study and revised the manuscript; Y W designed the study, analyzed the data, wrote and revised the manuscript, supervised and supported the study. All authors read and approved the final manuscript.
